# Methods for Optimizing Photoplethysmography Signal Quality

**DOI:** 10.3390/s26206669

**Published:** 2026-10-21

**Authors:** Oludotun Ode, David V. Anderson, Omer T. Inan

**Affiliations:** School of Electrical and Computer Engineering, Georgia Institute of Technology, Atlanta, GA 30332, USA; anderson@gatech.edu (D.V.A.); omer.inan@ece.gatech.edu (O.T.I.)

**Keywords:** DC offset cancellation, multi-channel data fusion, photoplethysmography (PPG), real-time calibration, seismocardiography (SCG), softmax function, wearable sensors

## Abstract

Although chest-mounted photoplethysmography (PPG) has proven useful in clinical applications, it nevertheless remains vulnerable to dynamic-range challenges. Because the chest is less well perfused than traditional PPG measurement sites, it yields weaker pulsatile signals that may be masked by large DC baselines. Some commercial sensors feature coarse built-in DC cancellation, but they inject considerable uncompensated artifacts. First, this paper presents a hardware–software codesign using a commercial sensor with a DC cancellation feature, which is overridden by smoothed, software-defined control inputs. A real-time calibrator estimates and cancels DC components using an exponentially weighted moving average. Second, a linear predictive coding-based artifact-elimination method is introduced. Third, a multi-channel data fusion method using a softmax combiner is developed to improve PPG signal quality. System performance was evaluated using experimental data from six human subjects in a seated laboratory environment. Results indicate that the methods remove DC offsets and step artifacts, producing waveforms that are similar to ground-truth references. Quantitative results also show that the proposed combiner achieved a signal-to-noise ratio of 8.47±3.14 dB on its composite waveform, providing modest improvements (p=0.031) compared to traditional equal-gain (7.91±2.86 dB) and maximal-ratio (8.03±2.87 dB) combiners.

## 1. Introduction

Photoplethysmography (PPG) is a well-established, noninvasive optical technique used to capture arterial blood volume pulse waveforms [1,2]. Historically, its primary clinical application has been measuring blood oxygen saturation, which is estimated from the AC (pulsatile) and DC (baseline) components of the PPG signal [3]. However, with the recent proliferation of wearable devices, including smartwatches for consumer health tracking, PPG has seen extensive adoption in continuous heart rate (HR) monitoring [4].

Beyond HR tracking, recent studies have demonstrated that PPG-derived features can enable physiological stress assessment and hemodynamic monitoring in heart failure patients [5,6]. To achieve this, a chest-mounted form factor is highly advantageous. This site enables simultaneous acquisition of PPG and seismocardiography (SCG) measurements by leveraging the SCG’s proximity to the heart, where cardiomechanical signals originate. Crucially, features extracted from the SCG waveform can be combined with PPG features to derive physiological biomarkers—such as pulse transit time (PTT). The main clinical interest in the PTT metric is that it serves as a surrogate for pulse wave velocity, which is a primary measure of arterial stiffness that can lead to cardiovascular diseases [7]. In addition, these combined PPG- and SCG-derived metrics have also been shown to be significant predictors for tracking cardiovascular response to mental stress [5].

### 1.1. Challenges of Chest-Mounted Photoplethysmography

Despite these advantages, deploying a PPG sensor on the chest presents significant technical challenges. First, sensor location heavily influences signal quality. Hertzman and Tur demonstrated that PPG signal characteristics vary dramatically over different anatomical sites [8,9]. Specifically, Tur characterized peripheral perfusion as a function of anatomical location, showing that the arterial blood supply to the trunk is substantially lower than that of highly perfused sites like the ears, hands, and fingers, at which traditional PPG measurements are taken [9].

Second, the backscattered light from blood vessels that is detected by reflectance-mode PPG (R-PPG)—the sensing modality used by chest-mounted PPG—is highly attenuated. Therefore, the pulsatile component of an R-PPG signal is typically very weak compared to its transmissive counterpart [2]. In many PPG applications (excluding oxygen saturation estimation), this AC component is the sole signal of interest. Consequently, a large DC baseline component unnecessarily limits the system’s performance by dominating the analog-to-digital converter (ADC) input and preventing sufficient amplification of the microvolt-level AC signal.

### 1.2. Prior Work in DC Mitigation

Several hardware and software architectures have been proposed to reduce the large DC offset in R-PPG systems. Toreyin et al. [10] developed an analog PPG front-end utilizing a distributed gain and bandpass filter architecture. This design carefully filters out the DC component prior to the main amplification stages, preventing signal saturation. However, this approach requires manual and precise analog tuning during signal acquisition, making it unsuitable for unobtrusive, user-friendly consumer applications where minimizing user interaction is critical. Furthermore, each additional analog component along the signal path inevitably introduces electronic noise, degrading the signal-to-noise ratio (SNR).

To resolve these limitations, Ganti et al. [11] proposed an architecture featuring a commercially available off-the-shelf (COTS) PPG sensor with programmable light-emitting diode (LED) currents. Their system continuously monitors the acquired signal amplitude and dynamically scales down the LED current when saturation is detected. Although this method prevents clipping and reduces the absolute DC offset, it also proportionally scales down the intensity of the backscattered light, consequently reducing the amplitude of the desired pulsatile AC signal.

Park et al. [12] developed a hardware DC cancellation feedback loop with a digital-to-analog converter (DAC) and a resistor. However, the reliance on custom hardware to handle DC offsets limits the translation of their method to applications using COTS sensors. Finally, the authors did not demonstrate how to remove the artifacts that would undoubtedly be introduced into the measured data with this method.

### 1.3. Proposed Architecture and Contributions

To overcome these limitations, this paper leverages the AFE4950 (Texas Instruments, Dallas, TX, USA), a highly configurable analog front-end (AFE) designed for synchronized acquisition of electrocardiogram (ECG) and PPG signals. The AFE4950 offers programmable system gains, adjustable LED currents, and a highly precise DAC that can inject or subtract a constant DC directly at the receiver input. This built-in DAC can be dynamically programmed to cancel the DC component of the signal measured by the photodiode (PD) without attenuating the AC signal [13].

The primary contributions of this paper are threefold:

#### 1.3.1. Real-Time Calibrator

This work presents an automated, real-time calibration algorithm for the AFE4950 that dynamically calculates and cancels the DC component of the PPG signal.

#### 1.3.2. Artifact Elimination

A digital signal processing framework is proposed to detect and eliminate transient artifacts introduced into the data stream during the dynamic DAC calibration steps.

#### 1.3.3. Multi-Channel Data Fusion Strategy

A multi-channel data fusion method is introduced to combine data from multiple PPG channels, providing a modest improvement in overall signal quality.

## 2. Materials and Methods

In previous work, an end-to-end data acquisition system was developed for measuring multimodal physiological data [14]. This system comprised analog PPG, ECG, and SCG circuits, with their respective signals sampled by an ADC on an MSP432P4111 MCU (Texas Instruments, Dallas, TX, USA). The acquired data were streamed to a custom Android application and subsequently transferred to cloud storage for post-processing.

**Figure 1:**
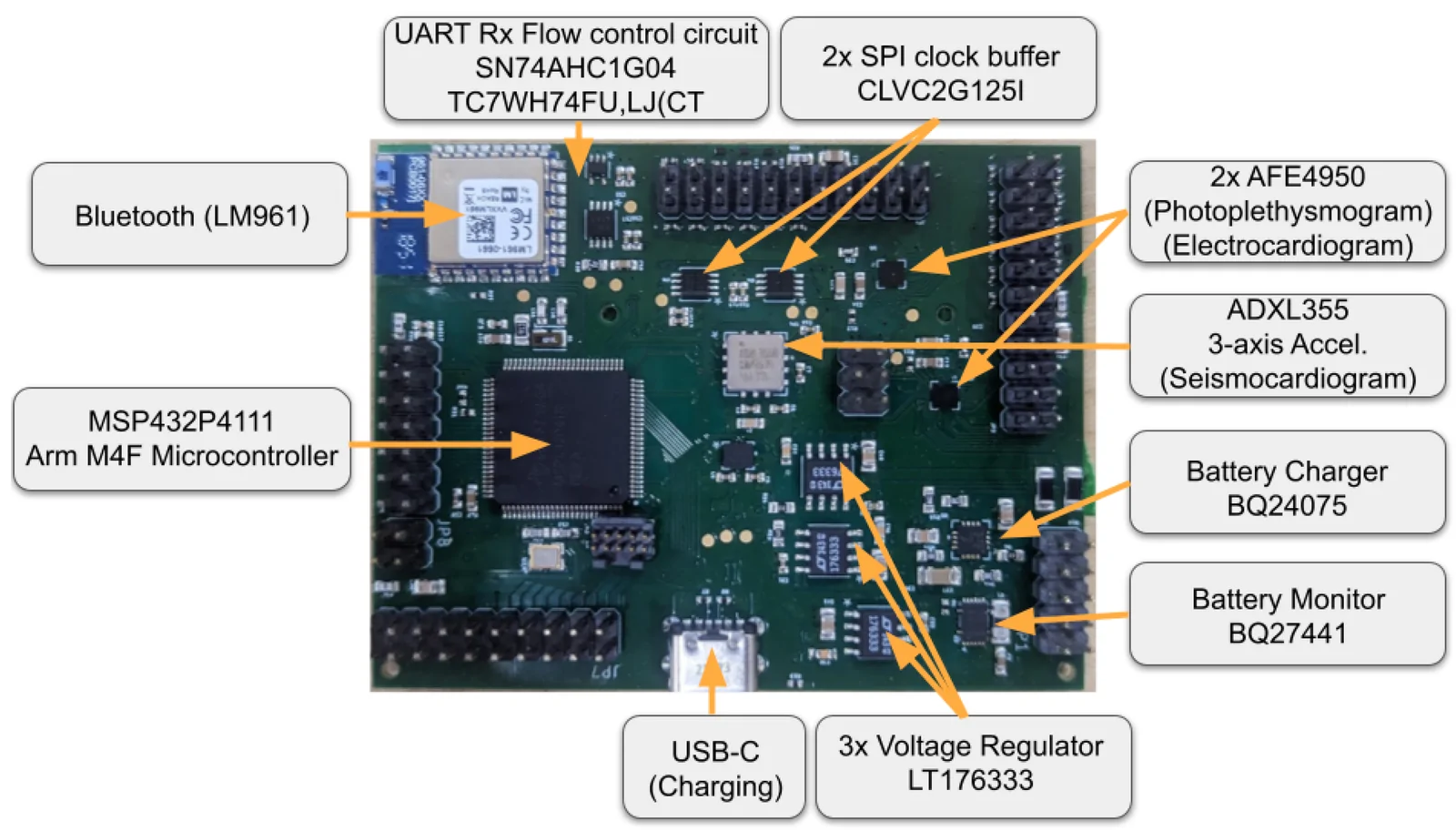
Hardware layout of the main PCB. Primary components include multimodal physiological sensors (PPG, SCG, ECG), a central microcontroller unit (MCU), and a Bluetooth transceiver. A dedicated USB-C interface is provided solely for battery charging.

**Figure 2:**
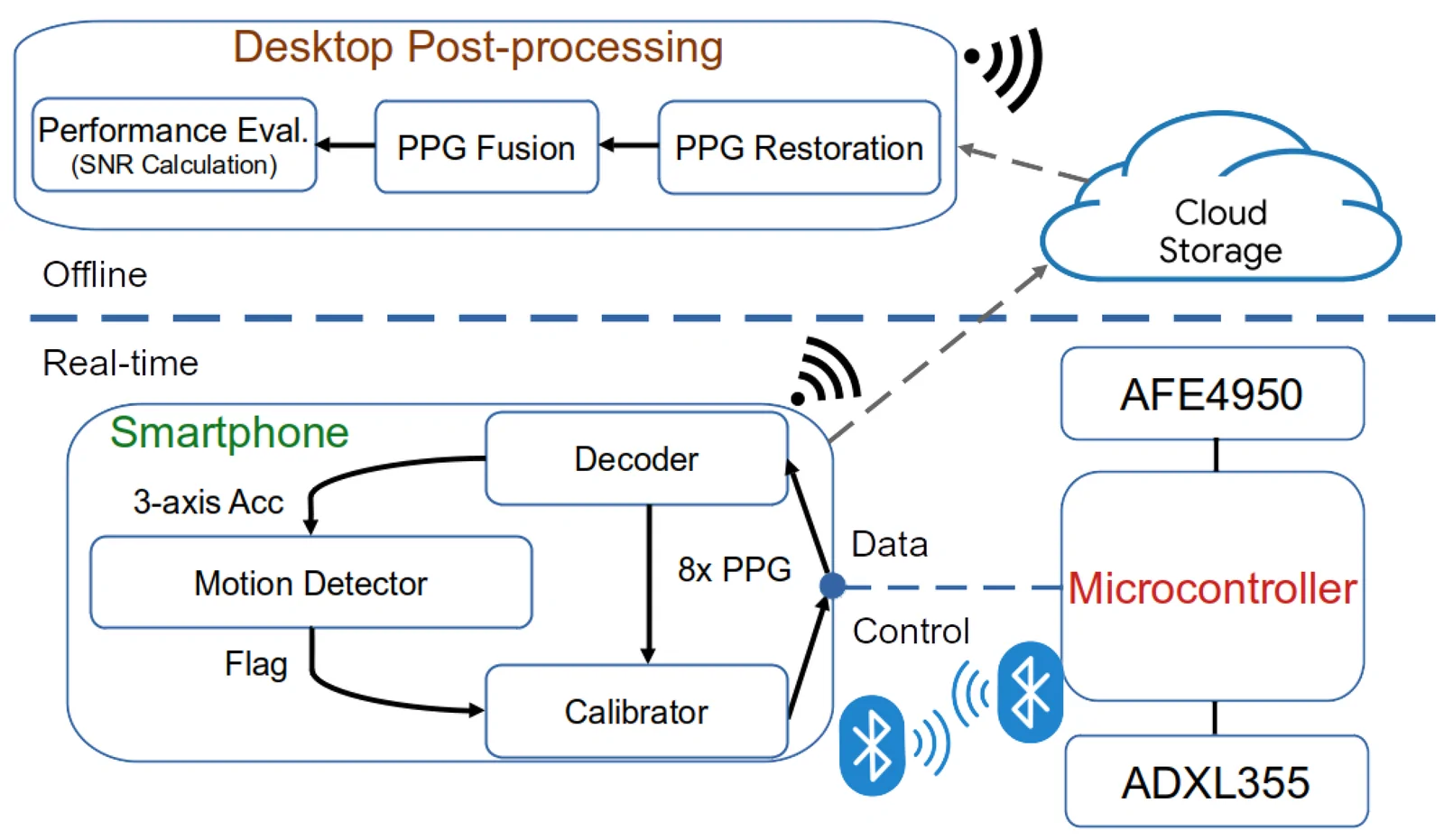
High-level overview of hardware–software architecture.

The system has since been updated to exclusively utilize COTS components as illustrated in Figure 1. Specifically, two AFE4950s are employed to acquire PPG and ECG signals, while an ADXL355 accelerometer (Analog Devices, Norwood, MA, USA) is used to measure SCG. To ensure synchronization, the hardware features of both sensors are leveraged to sample concurrently at a frequency of 512Hz. In total, one ECG channel, eight PPG channels, and three SCG axes are acquired. The PPG channels are configured with varying transimpedance amplifier (TIA) gains, LED driver currents, and active PD/LED states to comprehensively sweep the parameter space and demonstrate the signal fusion and reconstruction methods discussed in the following sections. Note that access to the AFE4950’s primary datasheet was provided under a Non-Disclosure Agreement, which precludes the publication of a hardware configuration table. The end-to-end data acquisition system is illustrated in Figure 2.

### 2.1. Motion Detection

The PPG signal is very susceptible to motion artifacts, which can distort the waveform and lead to sensor saturation. Therefore, the calibration procedure should be suspended during periods of significant motion. To detect these intervals, each accelerometer axis is partitioned into 500ms windows with 50% overlap. A motion artifact corruption condition is triggered if the signal power of the first difference of any partition, computed as

(1) $$P\left(a\right)=\frac{1}{N}\sum_{n=0}^{N-1}{\left(a_{n+1}-a_{n}\right)}^{2},$$

exceeds a threshold of $1.0\times 10^{-4}$ determined during system prototyping. The threshold was calibrated to tolerate low-amplitude postural shifts, though a validation of the motion detector’s sensitivity to ambulatory movements remains for future work. To reduce computational overhead, the data from each accelerometer axis were decimated by a factor of 10 before computing the signal power in (1).

### 2.2. Calibrator

The calibrator is implemented as a dedicated software thread running on the host smartphone. It executes at a 1Hz update rate, providing high responsiveness to slow baseline drifts while supporting rapid convergence of the feedback loop upon initialization. An exponentially weighted moving average (EWMA) of the raw PPG signal is computed to estimate its DC component, which is then used to adjust the DAC offset current of the AFE4950. The EWMA is a computationally efficient scheme for smoothing and local mean estimation in time-series data by progressively discounting historical samples relative to recent observations [15,16].

Let $V_{k}$ denote the $k^{th}$ sample of the measured PPG voltage and $\mu_{k}$ represent the corresponding EWMA estimate. At the $j^{th}$ calibration iteration, which occurs at sample index *n*, let *N* define the number of samples received since the last calibration update. The EWMA is updated recursively according to

(2) $$\mu_{k}=\alpha \mu_{k-1}+\left(1-\alpha \right)V_{k},\;\mathrm{for}\;k=n-N+1,\ldots ,n,$$

and the subsequent DAC register adjustment is formulated using the final estimate $\mu_{n}$ as

(3) $$i^{\left(j\right)}=i^{\left(j-1\right)}-\Delta i^{\left(j\right)},$$

where

(4) $$\Delta i^{\left(j\right)}≜\mathrm{sgn}\left(\mu_{n}\right)\left\lfloor \left|\frac{\mu_{n}}{\Delta V_{\min}}\right|\right\rfloor ,$$

and

(5) $$\Delta V_{\min}≜I_{\min}^{\mathrm{DAC}}G.$$

Here, *G* represents the gain of the TIA, and $I_{\min}^{\mathrm{DAC}}$ denotes the minimum resolvable increment of the DAC offset current. The parameter $i^{\left(j\right)}$ represents the discrete DAC register configuration, which is proportional to the physical compensation current. Following the calculation of the DAC offset current $i^{\left(j\right)}$, to improve the algorithm’s responsiveness, $\mu_{n}$ is modified to account for the expected change in the DC offset:

(6) $$\mu_{n}\leftarrow \mu_{n}-\Delta V_{\min}\Delta i^{\left(j\right)}.$$

This updated estimate serves as the initial state of $\mu_{k-1}$ in (2) at the onset of the ${\left(j+1\right)}^{th}$ calibrator iteration.

The EWMA is a discrete-time representation of a first-order, infinite impulse response low-pass filter [17]. Accordingly, the smoothing factor α is related to the physical system parameters by

(7) $$\alpha =\frac{f_{s}T-1}{f_{s}T+1},$$

where T=1 s is the filter time constant, selected to match the 1s update interval of the calibrator, and $f_{s}$ is the sampling frequency of the PPG signal. *T* can optionally be increased to make the calibrator even more insensitive to transients.

**Figure 3:**
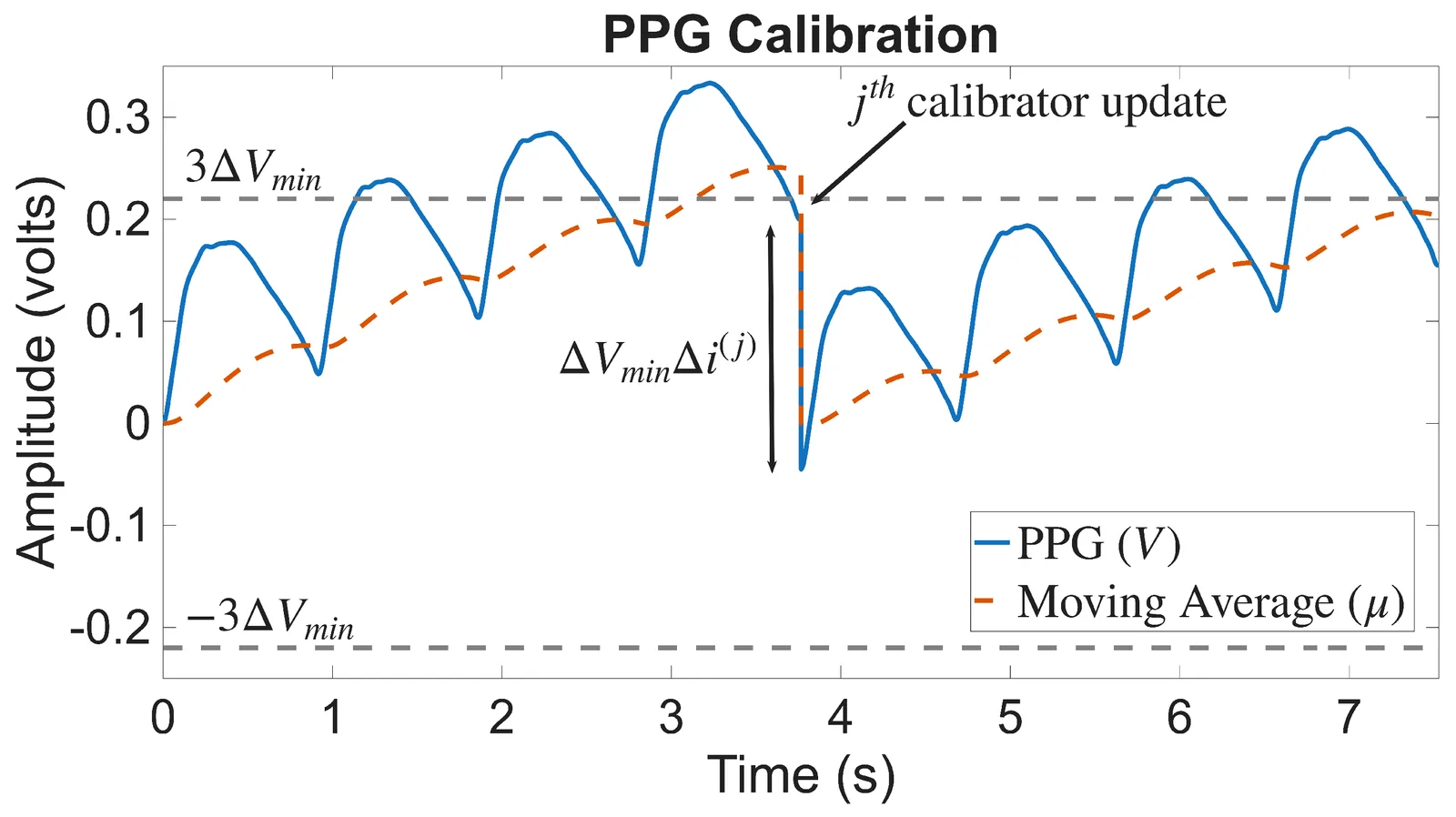
Simulated example of a PPG waveform with a slowly varying mean. No calibration updates are performed when the mean is within the dead-band region.

To prevent frequent, minor corrections due to transient baseline wander or respiration, the DAC register is updated only when the estimated DC component $\mu_{n}$ falls outside a dead-band threshold. This dead-band was chosen to be $\left[-3\Delta V_{\min},3\Delta V_{\min}\right]$. The minimum voltage change that can be effected by the DAC is $\Delta V_{\min}$, which, according to (5), is proportional to the receiver gain, *G*. For large *G*, $\Delta V_{\min}$ occupies a significant portion of the signal’s dynamic range. Therefore, using a larger multiple of $\Delta V_{\min}$ to set the dead-band region limits the calibrator’s flexibility. Figure 3 illustrates the calibration procedure using a simulated example.

### 2.3. Calibration Artifact Removal

The step-like adjustments of the calibration loop introduce severe high-frequency discontinuities. These artifacts disrupt downstream physiological signal processing algorithms, such as HR estimation and PTT calculation. To eliminate these discontinuities, an automated artifact-elimination pipeline was developed.

First, calibration points are identified via statistical outlier detection. This process is guided by two domain-specific assumptions: (1) the underlying PPG waveform is inherently band-limited to low frequencies, meaning nominal sample-to-sample variations are bounded, and (2) DAC transitions manifest as abrupt, isolated step functions. Thus, calibration events are isolated by applying outlier detection to the first difference of the raw signal. An outlier is defined as any difference exceeding 20 times the interquartile range (IQR) beyond the upper or lower quartiles:

(8) $$x\notin \left[Q_{1}-20\cdot \mathrm{IQR},\;Q_{3}+20\cdot \mathrm{IQR}\right].$$

**Figure 4:**
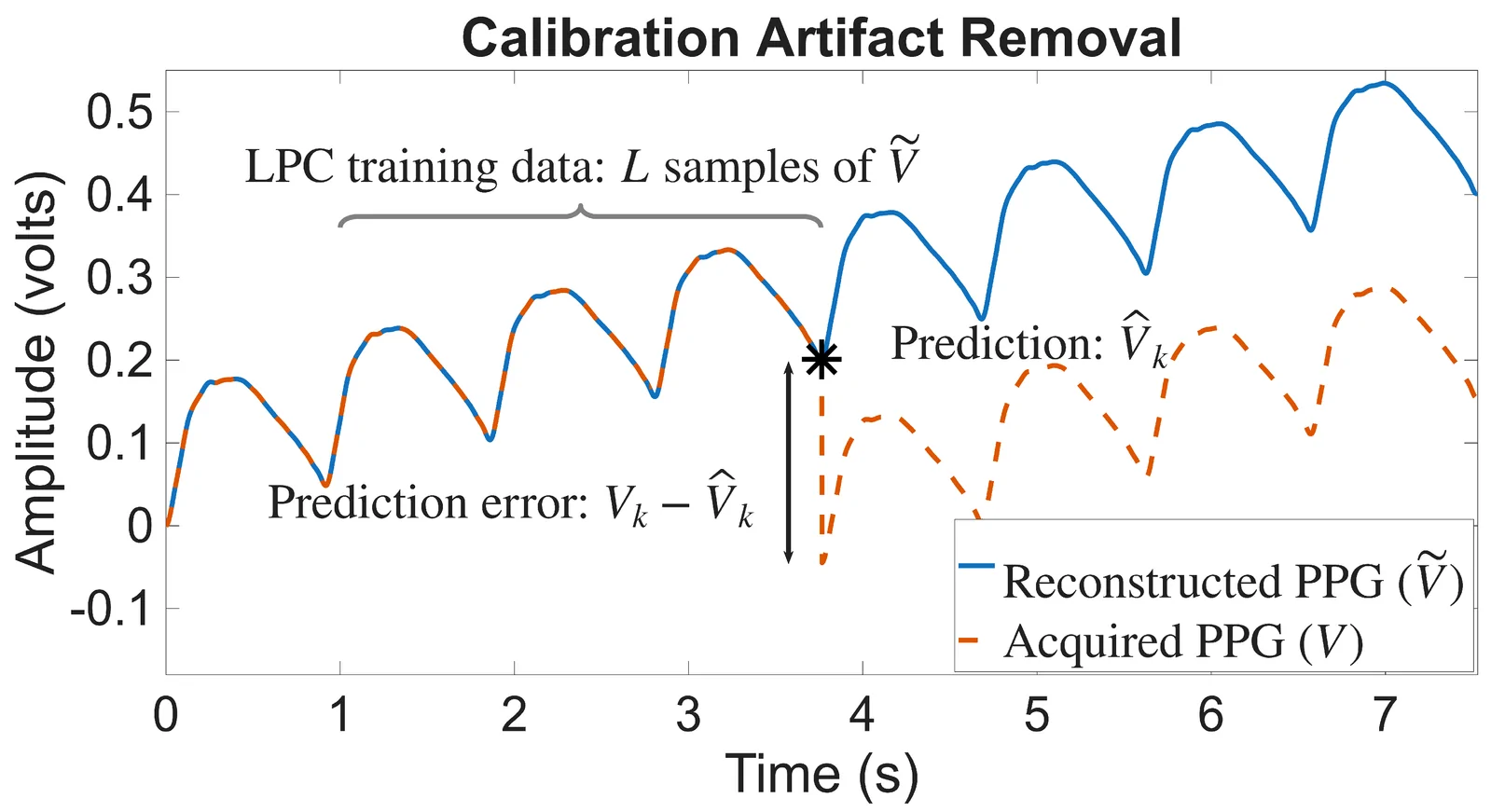
A simulation of the calibration artifact removal from the simulated waveform shown in Figure 3.

Second, let $\tilde{V}$ be the artifact-free reconstruction of *V*; at each index *k* where a calibration event is detected, a linear predictive coding (LPC) [18] model of order *p* is trained on the preceding artifact-free window ${\tilde{V}}_{k-L},\ldots ,{\tilde{V}}_{k-1}$. This model yields an estimated, uninterrupted signal value ${\hat{V}}_{k}$. The resulting prediction error, $\left(V_{k}-{\hat{V}}_{k}\right)$, is subtracted from every subsequent raw sample of *V* up until the next calibration event, neutralizing the DC step. For this implementation, the predictor order was set to p=9, and the training window length was set to *L* = 10,000 samples, as much smaller training windows were shown to yield substandard prediction accuracy during prototyping. Figure 4 simulates the signal restoration method used to suppress the artifacts from the example in Figure 3.

In principle, an alternative to the proposed signal restoration method would involve explicitly logging the sample index *m* at which each calibration update occurs, alongside the theoretical DC offset shift, $\Delta V_{\min}\Delta i^{\left(j\right)}$. However, capturing sample-accurate calibration timestamps would require substantial structural modifications to the embedded architecture in a manner that would severely constrain throughput and available signal bandwidth. To minimize communication overhead at high sampling rates, the ultra-low-power MCU uses the sensor’s hardware FIFO buffers, transferring data in high-efficiency bursts via direct memory access. This strategy offloads the MCU, preserving compute capacity for real-time tasks such as lossless data compression, lossless data communication, and handling variable-length messages from the host smartphone. Furthermore, calculating $\Delta V_{\min}$ relies on nominal TIA gains and DAC current step sizes, which inevitably deviate from physical values due to component manufacturing tolerances. By contrast, the proposed data-driven signal restoration technique is sensor-agnostic, eliminates reliance on nominal hardware specifications, and generalizes readily to alternative sensing architectures.

**Figure 5:**
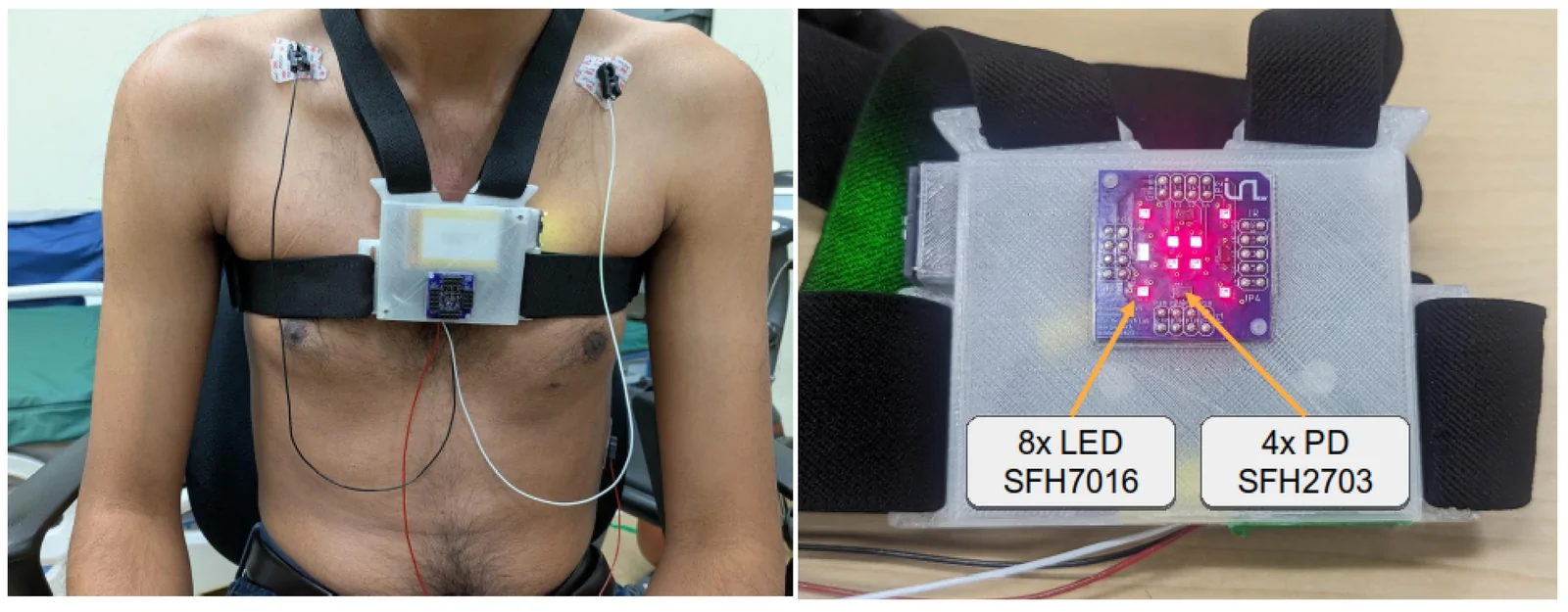
Mechanical assembly of the 3D-printed chest sensor package for the main PCB. A dedicated sternum board hosts an array of eight SFH7016 LEDs (Osram, Munich, Germany) and four SFH2703 PDs (Osram, Munich, Germany), which interface with a single AFE4950 on the main PCB via integrated connectors. The fully assembled enclosure is secured to the user’s chest with a strap.

Eight PPG channels are simultaneously acquired using the optical front-end on the sternum board depicted in Figure 5, sharing a common set of LEDs and PDs. To validate the calibration and reconstruction pipeline, PPG Channel 1 ($V^{1}$) was configured as a static ground truth for Channel 2 ($V^{2}$), with identical register settings, including LED drive currents, TIA gains, and active PD/LED states. Because both channels were configured with identical register settings, they undergo equivalent signal conditioning within the AFE4950’s time-multiplexed architecture. Combined with the shared optical front-end on the sternum board, the low-frequency nature of the PPG signal, and the fact that the channels are sampled sequentially within microseconds, the underlying physiological waveforms measured by both channels are very similar. The DAC current required to prevent saturation of the ground-truth channel was determined a priori and held constant throughout the recording. Because the remaining channels (3 through 8) were configured with varying parameters to sweep the search space, Channel 2 is the only channel with a direct static reference available for computing quantitative reconstruction error metrics.

Before evaluating reconstruction fidelity, a scalar offset $c^{*}$ was subtracted from the reconstructed signal to minimize the mean squared error relative to the ground truth:

(9) $$\begin{array}{cc} c^{*} & =\underset{c}{arg min}\frac{1}{2}{∥\left({\tilde{V}}^{2}-c\right)-V^{1}∥}_{2}^{2}\\ & =\frac{1}{N-n_{1}}\sum_{n=n_{1}}^{N-1}\left({\tilde{V}}_{n}^{2}-V_{n}^{1}\right), \end{array}$$

where the summation index $n_{1}$ is selected to exclude initial transient saturation epochs in ${\tilde{V}}^{2}$.

### 2.4. Multi-Channel Data Fusion

Following the classical diversity combining framework presented by Brennan [19], a composite signal f(t) can be constructed as a weighted linear combination of *C* input channels $f_{j}\left(t\right)$:

(10) $$f\left(t\right)=\sum_{j=1}^{C}a_{j}f_{j}\left(t\right).$$

Two diversity combining methods discussed in that paper are explored in this work:

1. *Equal-Gain Combining (EGC):* Assigns $a_{j}=1/C$ for all *j*, taking the simple unweighted average of all available channels.
2. *Maximal-Ratio Combining (MRC):* Weights each channel proportionally to its individual SNR profile:
  (11) $$a_{j}=\frac{{\mathrm{SNR}}_{j}}{\sum_{k=1}^{C}{\mathrm{SNR}}_{k}}.$$

Note that in this work, to ensure that variations in channel register settings did not disproportionately penalize the equal-gain combiner, the waveforms from all input channels $f_{j}\left(t\right)$ were amplitude-normalized before initiating the fusion procedures.

The metric ${\mathrm{SNR}}_{j}$ for each channel is estimated using a modified template-matching approach adapted from Inan et al. [20]. An ensemble template waveform is first constructed by averaging segmented, aligned cardiac cycles. Individual beats are then aligned with this template using matched filtering and scaled to minimize the residual Euclidean error. Letting $\sigma_{\mathrm{signal}}^{2}$ denote the variance of the scaled template and $\sigma_{\mathrm{noise}}^{2}$ represent the variance of the residual error, the channel quality is quantified as a linear ratio:

(12) $$\mathrm{SNR}≜\frac{\sigma_{\mathrm{signal}}^{2}}{\sigma_{\mathrm{noise}}^{2}}.$$

Based on the formulations in (11) and (12), modeling constraints dictate that the weights for the MRC evaluated in this work diverge slightly from those discussed in [19]. Crucially, both frameworks remain conceptually equivalent: they scale weights in proportion to a signal-strength metric and inversely to a noise metric.

In radio frequency communication, MRC is proven to be the mathematically optimal linear combining method for maximizing SNR under the assumption of independent, additive white Gaussian noise [19]. However, these assumptions do not strictly hold for PPG signals, where noise artifacts are highly correlated and often multiplicative (e.g., motion-induced coupling) [21,22]. Furthermore, in many applications, the main goal is the precise localization of morphological fiducial markers (such as the systolic foot) rather than maximizing global SNR. Constructing a composite signal using poorly performing channels can smooth out and degrade these important geometric markers.

To mitigate this, a mechanism is needed to aggressively penalize low-quality channels while preserving the relative contribution of high-quality inputs. The use of the softmax function [23] on the estimated channel SNRs is proposed to compute the fusion coefficients. The softmax combiner acts like a soft selection switch. When one channel has a substantially higher SNR, it selects that channel and mutes the others—mimicking the selection combiner—while distributing weights more evenly when the SNRs are comparable. This behavior is desirable in physiological monitoring. For instance, if beats in one channel contain morphological irregularities—which cannot be characterized as additive white Gaussian noise—while beats in another channel are smooth, combining them with linear weights would cause those irregularities to distort the composite signal. The weights for the softmax combiner are computed according to:

(13) $$a_{j}=\frac{\exp({\mathrm{SNR}}_{j})}{\sum_{k=1}^{C}\exp\left({\mathrm{SNR}}_{k}\right)}.$$

Note that in (11) and (13), ${\mathrm{SNR}}_{j}$ is evaluated directly as a linear ratio, rather than in decibels (dB).

### 2.5. Subject Demographics

Six subjects (five males, one female; age: 24.7 ± 3.1 years; height: 177.7 ± 6.5 cm; weight: 71.1 ± 15.3 kg; BMI: 22.3 ± 3.5 kg/m^2^; Fitzpatrick skin type: 5 Type III and 1 Type IV) participated in this study.

### 2.6. Physiological Measurements

A custom 3D-printed sensor package housing the main PCB (Figure 1) was secured to the subject’s chest, positioning the ECG electrodes in a Lead II configuration (Figure 5). One AFE4950 sensor simultaneously acquired eight PPG channels, while a second AFE4950 was dedicated to ECG acquisition. Concurrently, an ADXL355 sensor recorded 3-axis accelerometer data. The measurements were recorded from seated participants in a laboratory setting under resting conditions, subject only to natural respiration and low-amplitude postural sway.

**Figure 6:**
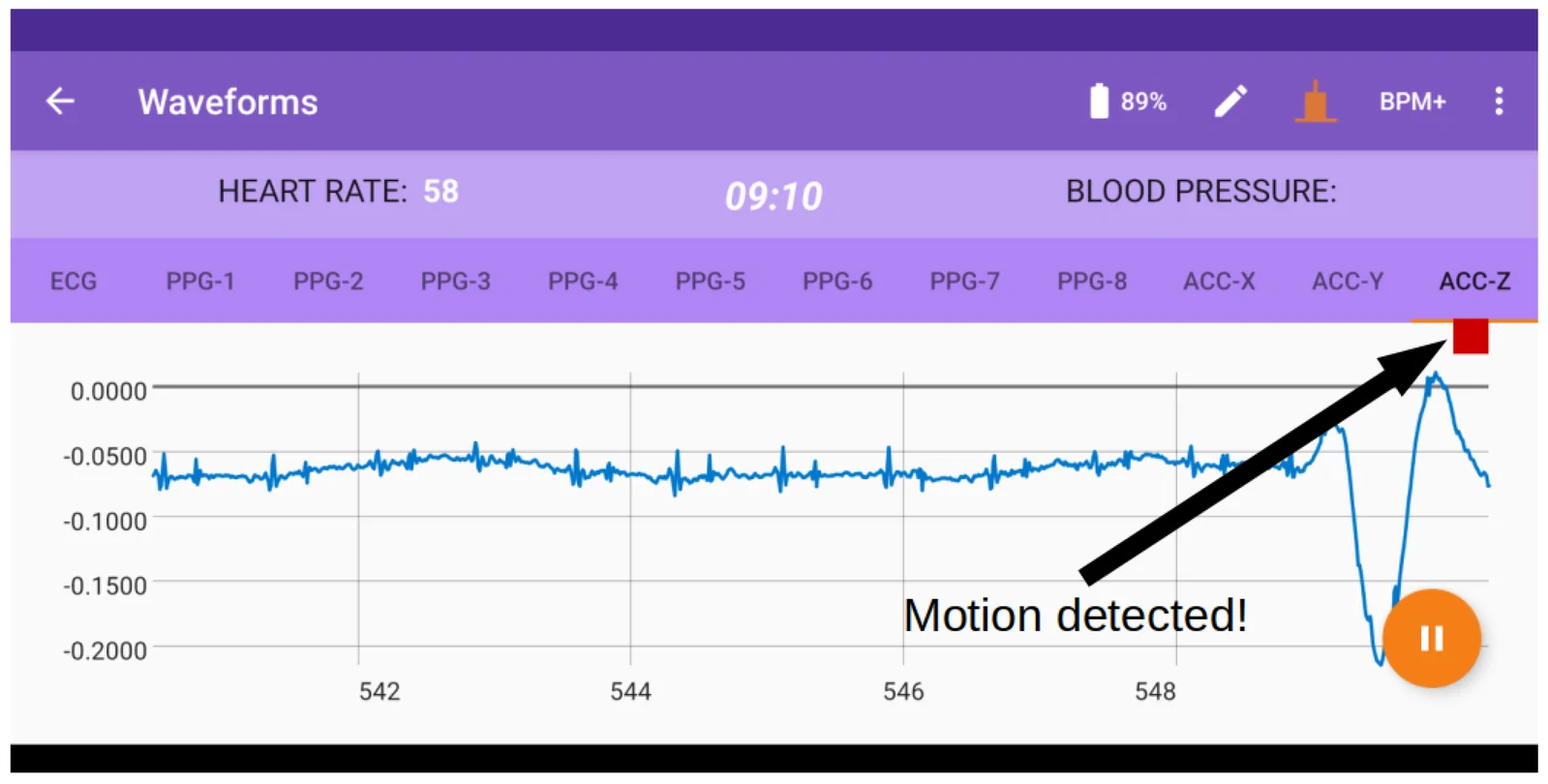
Real-time Android user interface displaying continuous background data streaming via the GraphView^®^ library. The dorso-ventral SCG waveform clearly delineates individual cardiac beats. When the algorithm detailed in Section 2.1 detects motion, the interface suspends calibration updates and changes the indicator status to red.

Before starting the data recording, the DAC current required to cancel the offset current for Channel 1 was determined and manually programmed into the corresponding AFE4950 register. This step was performed via a smartphone graphical user interface (Figure 6) exposing the registers for all PPG channels. Five minutes of data were then streamed from the device to the smartphone, utilizing the real-time calibration algorithm to automatically cancel the DC component of the acquired PPG signals for channels 2 through 8 by sending calibration commands over Bluetooth. Finally, the measurements were saved to cloud storage and extracted for post-processing and analysis in MATLAB 2025a (MathWorks, Natick, MA, USA), which included signal restoration and multi-channel data fusion.

## 3. Results and Discussion

### 3.1. Calibration and Artifact Removal

**Figure 7:**
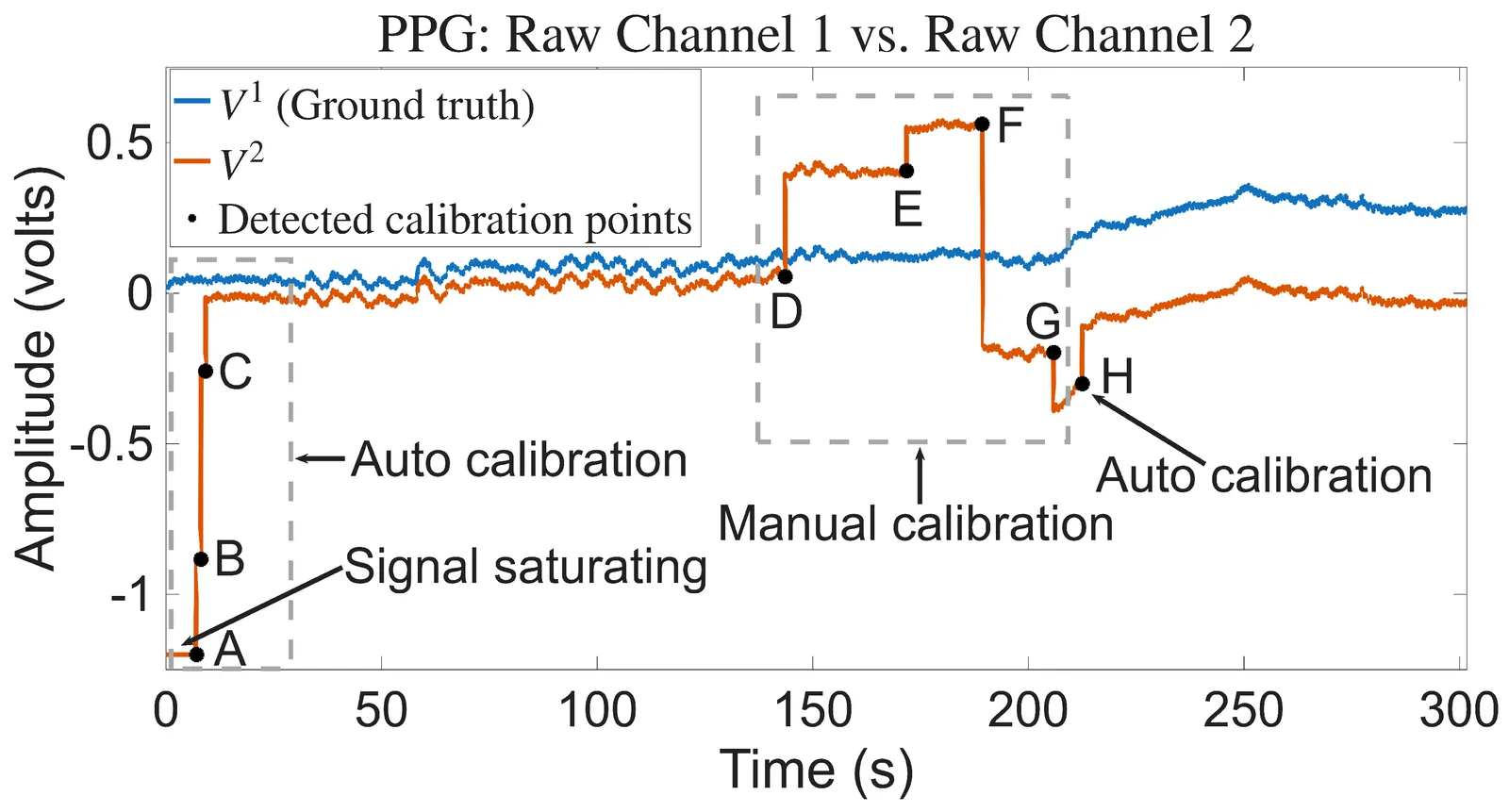
The calibrator automatically cancels the initially large DC offset in Channel 2, but significant artifacts are present in the data. The outlier detection method correctly identifies all eight calibration events, annotated A–H in the figure.

**Figure 8:**
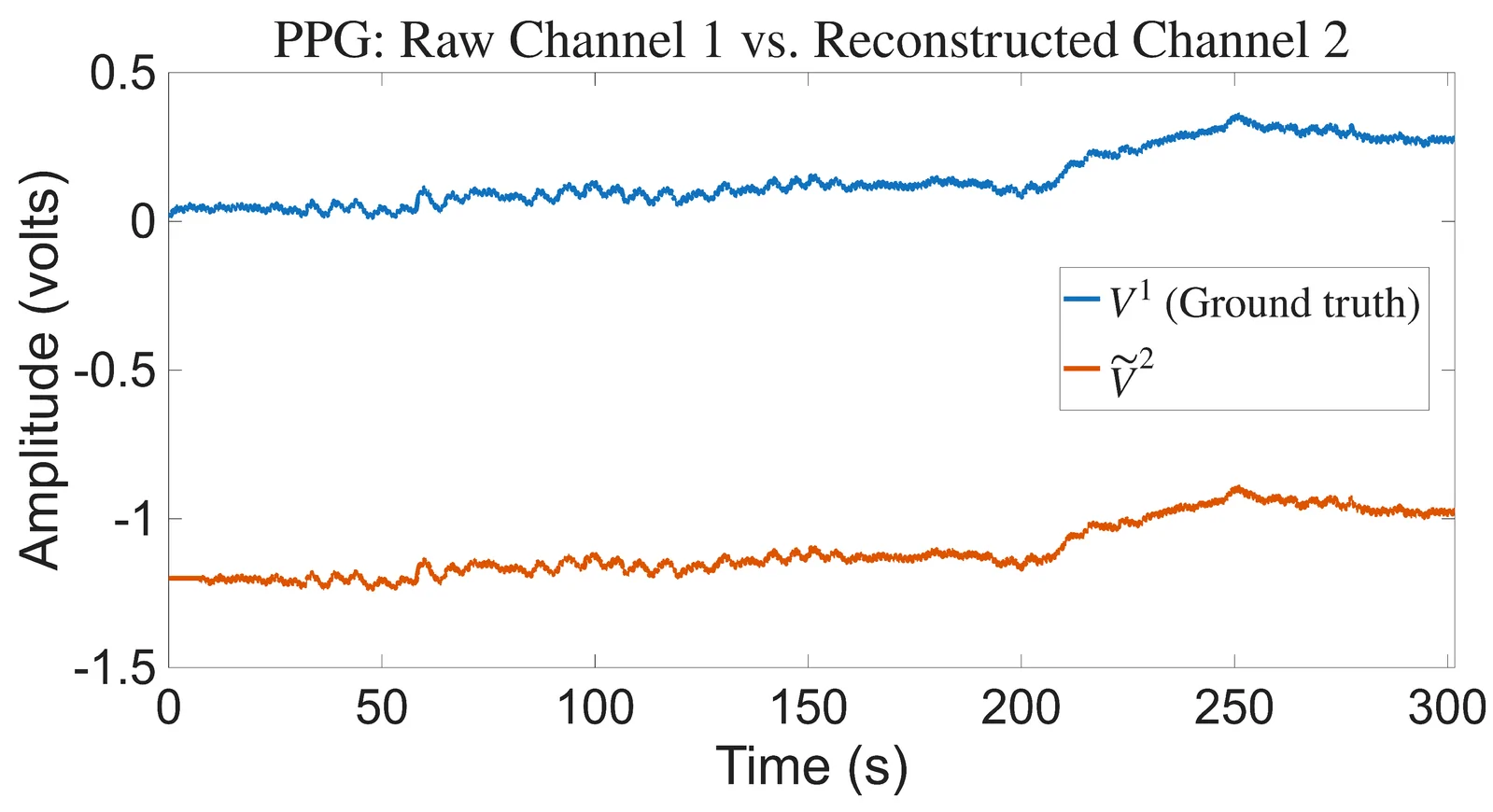
Raw PPG for Channel 1 vs. reconstructed PPG for Channel 2. ${\tilde{V}}^{2}$ is similar to $V^{1}$, but with a DC offset.

**Figure 9:**
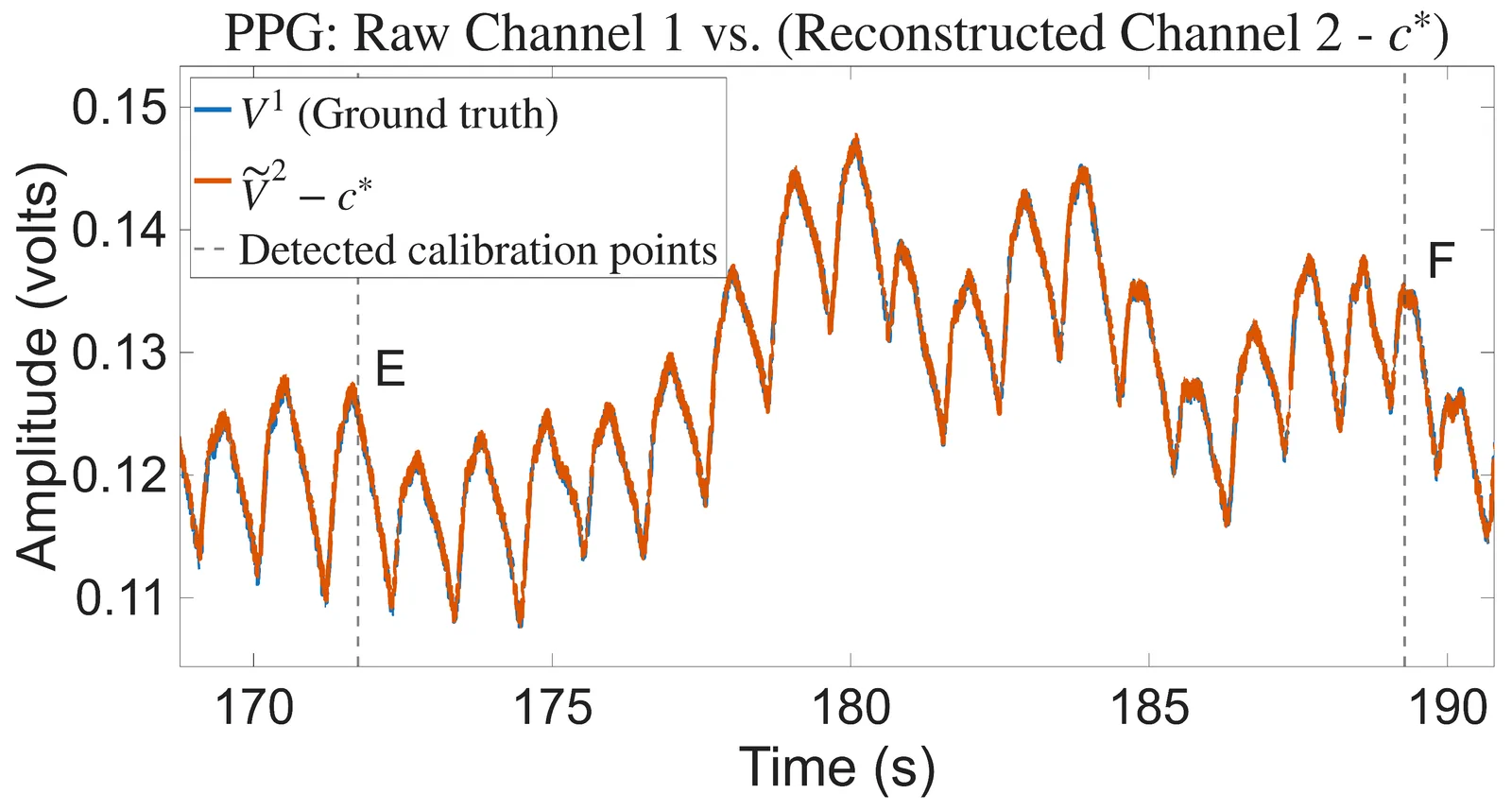
Raw PPG for Channel 1 vs. reconstructed PPG for Channel 2. ${\tilde{V}}^{2}$ is a copy of $V^{1}$ after subtracting the average error between the waveforms in Figure 8.

Figure 7, Figure 8 and Figure 9 are representative results of the calibration and signal restoration process, which demonstrate the proposed method’s capability to automatically cancel large DC offsets and eliminate transient calibration artifacts, resulting in a reconstructed waveform that closely tracks the reference signal. In addition, they indicate that the algorithm suppresses spurious calibration updates triggered by minor baseline drifts. Complementing this, Figure 6 illustrates the system’s ability to suspend calibration updates during periods with considerable motion artifacts.

The voltage range of the PPG sensor is ±1.2 V; thus, the waveform in Figure 7 initially saturates at –1.2 V. There is a noticeable delay of seven seconds before the first observable change in the DC offset at the point labeled A. This occurs because signal saturation prevents the calibration algorithm from accurately estimating the DAC current required to cancel the DC offset. Furthermore, the EWMA estimate is updated to reflect the expected changes in the DC offset immediately preceding the calibration update command. This improves responsiveness during normal signal acquisition but impedes algorithm convergence when the signal is saturating. However, once the signal is within range, the algorithm requires only two additional iterations (i.e., two seconds) to converge and effectively cancel the initially large DC offset. To more clearly illustrate the efficacy of the calibration and artifact removal processes, four additional changes were artificially made to the DAC current at the points labeled D, E, F, and G, with the auto-calibrator disabled (an additional DAC update occurred at H, but this was performed with the auto-calibrator re-enabled). In Figure 7, a noticeable baseline deviation is observed beginning right after G, corresponding to a minor postural adjustment. This highlights two key properties of the system: (1) the auto-calibrator still triggers at H, overlapping with the change in posture, to correct the offset injected by manual calibration, which means that the motion detector tolerated this incidental low-amplitude postural change without unnecessarily suspending the calibration process; and (2) a further elevation of the baseline (+0.16 V) did not result in a calibration update, confirming that the calibrator ignores minor baseline drifts that fall within the configured dead-band region. The plot also demonstrates that the outlier detection method described in Section 2.3 correctly identifies all eight calibration locations A–H on the waveform for Channel 2.

Figure 8 plots the PPG data for Channel 1 (the reference channel) alongside the artifact-free reconstruction of the Channel 2 data. A large DC offset separates the waveforms because the reconstruction algorithm assumes that the samples preceding the calibration location are artifact-free and subsequently employs them as estimators for the sample at the calibration point. At initialization, these artifact-free samples saturate at –1.2 V, thereby centering the resulting reconstruction around –1.2 V. Note that because this baseline shift exists purely as a digital representation in software, it poses no risk of dynamic-range clipping. The resulting baseline offset will subsequently be eliminated during standard downstream PPG pre-processing with bandpass filtering.

Because the hardware in this study was configured to sample at 512 Hz to synchronize with the SCG and ECG sensors, the acquired PPG waveforms are highly oversampled. As a result, the most recent sample consistently accounted for nearly 100% of the predictor weight across all subjects. At such high sampling rates, a simple sample-and-hold estimator may replace the LPC model. This simple estimator has an O(1) complexity and requires only one memory access, yielding a highly computationally efficient alternative for resource-constrained systems. However, the full LPC framework is presented here as a general solution for wearable systems. In many ultra-low-power applications, PPG is sampled at much lower rates (e.g., 25–50 Hz) to conserve battery life. At these lower rates, a sample-and-hold approach may be inadequate for tracking the signal trajectory during rapid morphological changes, such as the systolic upstroke. In that scenario, the LPC model is required to accurately capture the data dynamics. Although the LPC method is computationally expensive, it can be implemented more efficiently using the Levinson–Durbin recursive algorithm, which has an O(Lp) complexity for computing the autocorrelation. In addition, it is reasonable to expect that the number of samples needed to represent the same physiological time window shrinks proportionally at a lower sampling rate. This would reduce the training window length *L*, thereby significantly decreasing the computational cost and ensuring the full artifact-removal method remains viable even on low-power systems.

**Figure 10:**
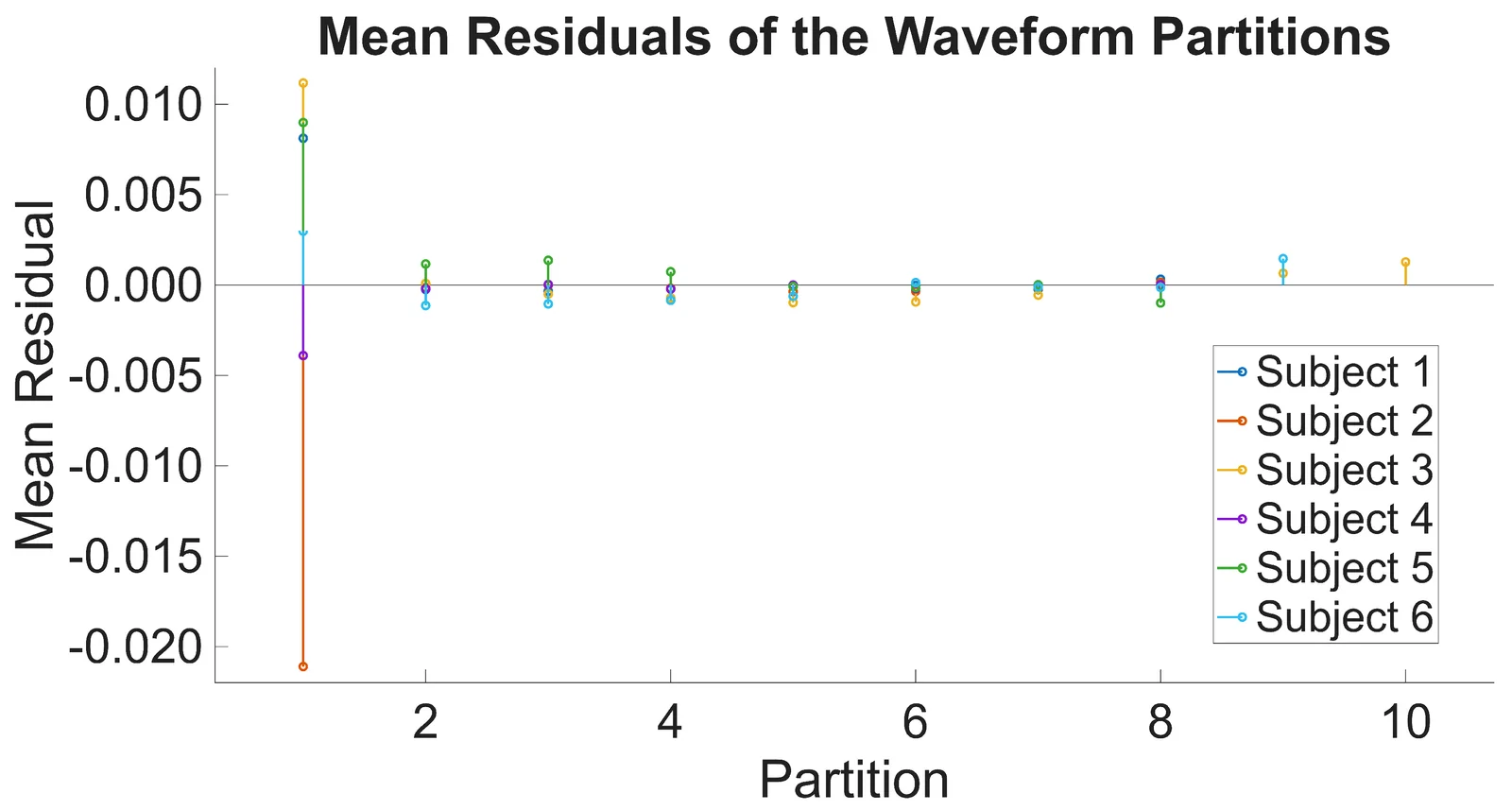
The calibration events partition the residual $\left({\tilde{V}}^{2}-c^{*}\right)-V^{1}$, and the mean of each partition is shown. The first partition corresponds to the saturated waveform, which drives the large amplitude.

Figure 9 displays magnified views of the reference waveform and the reconstructed Channel 2 PPG signal after subtracting the average error between the waveforms plotted in Figure 8. Both waveforms closely track each other, despite the manually injected calibration artifacts in the Channel 2 PPG data at E and F. To assess the signal reconstruction fidelity, the normalized root-mean-square error (NRMSE) and the Pearson correlation coefficient were computed between the reconstructed waveform (${\tilde{V}}^{2}$) and the ground truth ($V^{1}$) for all six subjects, as reported in Table 1. Note that both metrics were calculated over the entire recordings, including the region where the waveforms saturated. Compared to the reference waveforms, the reconstructed versions achieved a very high Pearson correlation (0.9940 to 0.9998) and low NRMSE (0.62% to 1.91%). To evaluate the residual step error per calibration event, the residual $\left({\tilde{V}}^{2}-c^{*}\right)-V^{1}$ was partitioned by the calibration events, and the mean of each partition was computed. Figure 10 shows that the errors are small, with most of the values close to zero (excluding the first residual, which is associated with the saturated waveform).

### 3.2. Sensor Fusion

**Figure 11:**
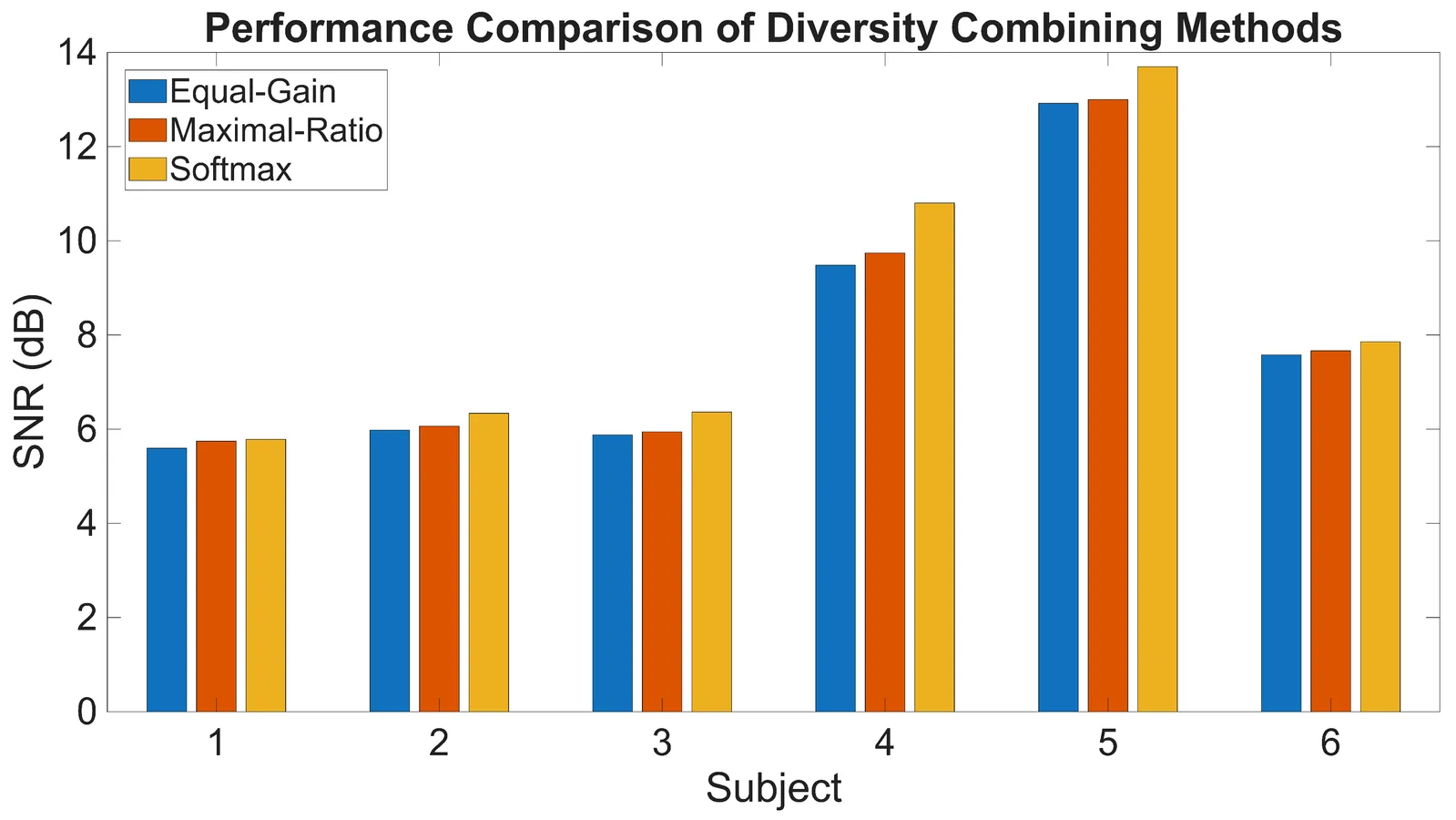
Bar chart showing the SNRs for the composite waveforms obtained with the combiners discussed in this paper.

Figure 11 and Table 2 display the computed SNR values and paired differences for the composite waveforms generated by each combining method. These values were calculated using the entire 5-min recording. The initial saturation phase was intentionally retained in the calculation to provide a real-world assessment of the system’s performance from a cold start. Over this full window, the equal-gain, maximal-ratio, and softmax combiners yielded average SNRs of 7.91±2.86, 8.03±2.87, and 8.47±3.14(dB), respectively. A Wilcoxon signed-rank test confirmed that the softmax combiner yielded a statistically significant improvement in SNR compared to both the equal-gain and maximal-ratio combiners (N=6, W=0, p=0.031). The softmax combiner produced the highest SNR waveforms across all six subjects. Moreover, even with Subject 1, the softmax combiner still yielded a marginally higher SNR (+0.04 dB) than the maximal-ratio combiner. The equal-gain combiner—a straightforward strategy equivalent to simple averaging—consistently exhibited the lowest overall SNRs among the evaluated methods across all subjects. Although the absolute SNR of the composite waveforms (averaging 8.47 dB) is lower than values typically observed at highly perfused sites like the fingertip, it shows the challenge associated with measuring high-quality R-PPG at the chest. In this low-SNR regime, the softmax combiner’s ability to extract improvements over traditional methods is very desirable for continuous wearable monitoring.

**Figure 12:**
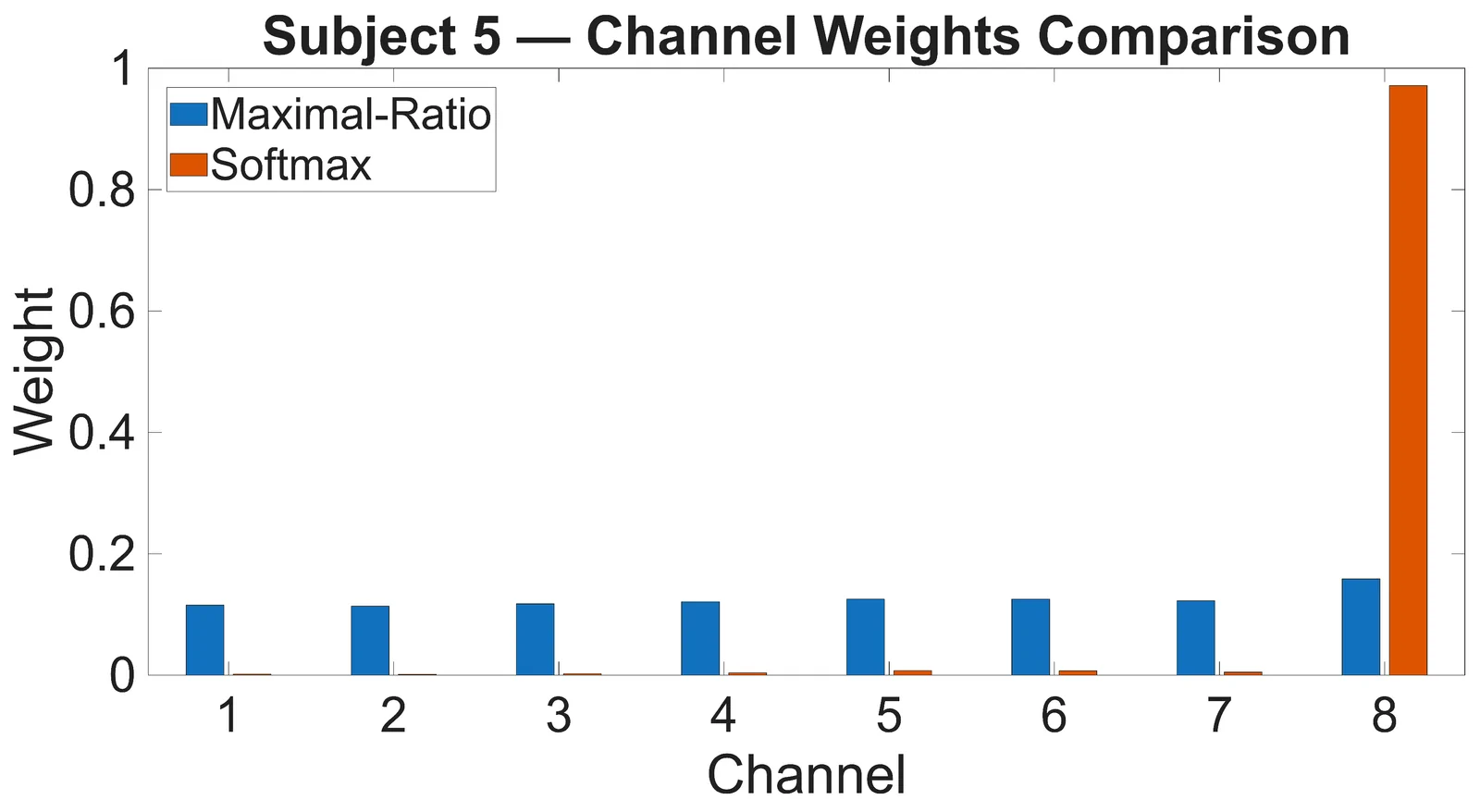
Bar chart showing the maximal-ratio and softmax fusion weights for each of the eight channels that were measured for Subject 5.

**Figure 13:**
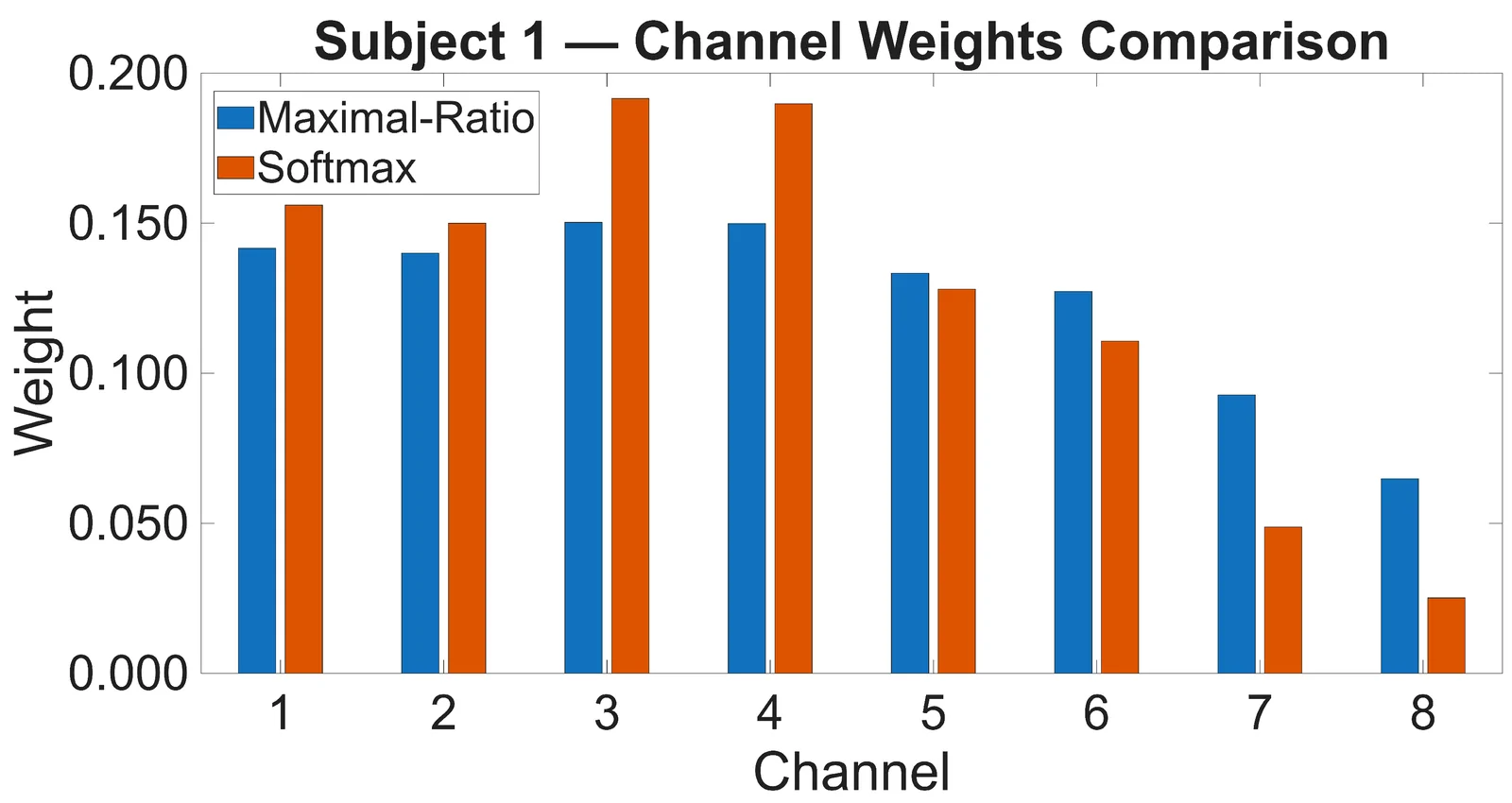
Bar chart showing the maximal-ratio and softmax fusion weights for each of the eight channels that were measured for Subject 1.

Figure 12 illustrates the channel weights computed by the maximal-ratio and softmax combiners for Subject 5. The maximal-ratio combiner distributes weights relatively evenly across the eight channels, though the weight for Channel 8 (0.16) remains marginally higher than the rest. By contrast, the softmax combiner concentrates its weight distribution unevenly, assigning a markedly high weight of 0.97 to Channel 8. Conversely, Figure 13 reveals much less pronounced weight discrepancies for both combiners on Subject 1. This variation in weight distribution between Subjects 1 and 5 under the softmax combiner is due to the spread of SNRs across the channels. Because the softmax function is shift-invariant, its weight distribution is determined by the spread of channel SNRs rather than their absolute magnitudes. For Subject 1, the signal quality was uniformly poor across all channels, resulting in a narrow spread of SNRs, which caused the exponential function’s non-linearity to enforce a highly conservative blending behavior. Conversely, for Subject 5, a wider spread caused the softmax combiner to allocate nearly all weight to the primary channel, effectively selecting the channel with the maximum SNR. The capacity of the softmax combiner to adapt to the SNR spread—remaining conservative when channel differences are minimal, while optimizing aggressively when clear differences exist—results in an improvement over the other combiners.

### 3.3. Limitations

Several limitations of this study should be noted. The experiments were conducted on a small, young, and relatively homogeneous cohort (N=6). Age-related changes in vascular compliance could change the waveform morphology and baseline SNR, which may affect the softmax combiner’s weights. Also, the experiments were performed in a controlled laboratory environment, with subjects in a seated, resting posture. Consequently, the signal restoration and data fusion algorithms have not yet been validated under conditions of ambulatory motion or diverse physical activities. Under strong motion artifacts, the motion detector would likely suspend the calibrator. However, because intense physical activity corrupts the underlying pulsatile PPG signal, suspending calibration during these intervals is required to prevent the system from adapting to noise. In addition, variables known to influence the PPG signal—specifically, the mechanical contact pressure (strap tension) of the sensor package against the chest [24] and chest adipose tissue thickness, which can attenuate the signals and make DC calibration even more important—were not quantified or controlled during these experiments. Furthermore, although the physiological data were acquired using embedded hardware, the signal restoration and multi-channel data fusion pipelines were performed offline using MATLAB rather than in real time on the microcontroller. Finally, although the softmax combiner yielded statistically significant improvements in SNR within this study, the sample size is small, and the SNR gains were marginal for some subjects. More validation in a larger, more diverse population is needed to determine how these improvements generalize.

## 4. Conclusions and Future Work

This work improves continuous R-PPG by introducing a real-time calibration method to optimize signal quality. The proposed hardware–software codesign, along with offline signal restoration and multi-channel data fusion algorithms, has broad utility across a wide range of downstream monitoring applications, including continuous physiological stress assessment and noninvasive hemodynamic tracking in patients with heart failure. In particular, experimental results demonstrate that the proposed calibration method improves PPG signal quality by canceling the large DC offset in the measured waveforms. At the same time, the offline processing performed in MATLAB suppresses transient calibration artifacts. Furthermore, the results demonstrate that the softmax combiner can combine signals from multiple PPG channels, with a performance improvement over traditional combiners.

Future work will investigate methods for automatically optimizing the remaining PPG sensor parameters. For each PPG channel acquired by the AFE4950 sensor, the LED current for each of the eight LEDs, the receiver gain, and the DAC offset current can be independently set via register settings. In addition, each of the eight LEDs and the four PDs can be independently enabled. This results in a very large search space over which to optimize the parameters controlling signal quality. To simplify this task, the current study selected the DAC offset current as the sole optimization parameter. This approach is valid because once the signal saturation problem caused by a large DC offset is mitigated, other parameters can be optimized more easily. For example, all LEDs and PDs can be enabled, and the LED currents and receiver gains can be set to large values, with the knowledge that the calibration routine presented in this paper can reduce the risk of saturation. However, care must be taken when automatically optimizing the remaining PPG sensor parameters. In particular, the DAC’s minimum voltage resolution ($\Delta V_{\min}=I_{\min}^{\mathrm{DAC}}G$) scales with the TIA gain. Therefore, gain changes must ensure that the minimum DAC correction steps do not become too coarse. In addition, real-time versions of the data fusion and artifact removal algorithms, currently developed in MATLAB, will be implemented. Finally, future evaluations will incorporate derivative-based morphological indices, such as PPG acceleration, to further validate the waveform reconstruction method.

## Figures and Tables

**Table 1:** Reconstruction performance metrics (Pearson correlation coefficient *r* and normalized root-mean-square error (NRMSE)) between the ground-truth reference ($V^{1}$) and the reconstructed signal (${\tilde{V}}^{2}$) across all six subjects (N=6).

Subject	Pearson Correlation (*r*)	NRMSE (%)
1	0.9940	1.91
2	0.9956	1.61
3	0.9994	0.86
4	0.9994	0.73
5	0.9998	0.62
6	0.9982	1.63

**Table 2:** Comparison of composite signal-to-noise ratios (SNR) and paired differences among Equal-Gain (EGC), Maximal-Ratio (MRC), and Softmax combining methods across six subjects (N=6).

	Absolute SNR (dB)			Paired Differences (dB)		
Subject	EGC	MRC	Softmax	MRC-EGC	Softmax-EGC	Softmax-MRC
1	5.60	5.74	5.78	+0.15	+0.18	+0.04
2	5.98	6.06	6.34	+0.08	+0.36	+0.27
3	5.88	5.94	6.36	+0.06	+0.48	+0.42
4	9.48	9.74	10.80	+0.26	+1.32	+1.06
5	12.92	13.00	13.70	+0.08	+0.77	+0.70
6	7.58	7.66	7.86	+0.09	+0.28	+0.19

**Note:** Wilcoxon signed-rank tests confirmed that the Softmax combiner yielded a statistically significant improvement in SNR compared to both the EGC method (N=6, W=0, p=0.031) and the MRC method (N=6, W=0, p=0.031). The MRC method also yielded significantly higher SNRs than the EGC method (N=6, W=0, p=0.031).

## Data Availability

The experimental data presented in this study, along with the MATLAB source code used for signal restoration and data fusion, are publicly available. The dataset can be accessed via the Georgia Tech Repository at https://doi.org/10.35090/gatech/82290 (accessed on 16 October 2026). The accompanying codebase is hosted on GitHub at https://github.com/oludotun-o/ppg_calib (accessed on 16 October 2026).

## Abbreviations

The following abbreviations are used in this manuscript:

ADC	Analog-to-digital converter
AFE	Analog front-end
COTS	Commercially available off-the-shelf
DAC	Digital-to-analog converter
ECG	Electrocardiogram
EGC	Equal-gain combining
EWMA	Exponentially weighted moving average
HR	Heart rate
IQR	Interquartile range
LED	Light-emitting diode
LPC	Linear predictive coding
MCU	Microcontroller unit
MRC	Maximal-ratio combining
PCB	Printed circuit board
PD	Photodiode
PPG	Photoplethysmography
PTT	Pulse transit time
R-PPG	Reflectance-mode photoplethysmography
SCG	Seismocardiography
SNR	Signal-to-noise ratio
TIA	Transimpedance amplifier

## References

[1] Allen J. (2007). Photoplethysmography and its application in clinical physiological measurement. Physiol. Meas..

[2] Park J., Seok H.S., Kim S.S., Shin H. (2022). Photoplethysmogram Analysis and Applications: An Integrative Review. Front. Physiol..

[3] Chatterjee S., Kyriacou P.A. (2019). A Monte Carlo Analysis of Optical Interactions in Reflectance and Transmittance Finger Photoplethysmography. Sensors.

[4] Castaneda D., Esparza A., Ghamari M., Soltanpur C., Nazeran H. (2018). A review on wearable photoplethysmography sensors and their potential future applications in health care. Int. J. Biosens. Bioelectron..

[5] Nawar A., Gazi A.H., Chan M., Sanchez-Perez J.A., Rahman F.N., Ziegler C., Daaboul O., Haddad G., Al-Abboud O.A., Ahmed H. (2026). Quantifying the Cardiovascular Response to Mental Stress Using a Compact Multimodal Wearable Sensing Patch. ACM Trans. Comput. Healthc..

[6] Klein L., Fudim M., Etemadi M., Gordon R., Tibrewala A., Hernandez-Montfort J., McCann P., Zier L., Shah K., Rollins A. (2025). Noninvasive Pulmonary Capillary Wedge Pressure Estimation in Heart Failure Patients With the Use of Wearable Sensing and AI. JACC Heart Fail..

[7] Diana G., Scardulla F., Puleo S., Pasta S., D’Acquisto L. (2025). Non-invasive estimation of arterial stiffness using photoplethysmography sensors: An in vitro approach. Sensors.

[8] Hertzman A.B. (1938). The blood supply of various skin areas as estimated by the photoelectric plethysmograph. Am. J. Physiol..

[9] Tur E., Tur M., Maibach H.I., Guy R.H. (1983). Basal Perfusion of the Cutaneous Microcirculation: Measurements as a Function of Anatomic Position. J. Investig. Dermatol..

[10] Töreyin H., Javaid A.Q., Ashouri H., Ode O., Inan O.T. Towards ubiquitous blood pressure monitoring in an armband using pulse transit time. Proceedings of the 2015 IEEE Biomedical Circuits and Systems Conference (BioCAS 2015).

[11] Ganti V.G., Carek A.M., Nevius B.N., Heller J.A., Etemadi M., Inan O.T. (2021). Wearable Cuff-Less Blood Pressure Estimation at Home via Pulse Transit Time. IEEE J. Biomed. Health Inform..

[12] Park J., Yang S., Kim H.C. (2019). Cuffless and Continuous Blood Pressure Monitoring Using a Single Chest-Worn Device. IEEE Access.

[13] Texas Instruments AFE4950 Ultra-Small, Integrated AFE for Wearable Optical Heart-Rate Monitoring, SpO2 and Electrical Bio-Sensing Data Sheet. https://www.ti.com/lit/ds/symlink/afe4950.pdf.

[14] Ode O., Orlandic L., Inan O.T. (2020). Towards Continuous and Ambulatory Blood Pressure Monitoring: Methods for Efficient Data Acquisition for Pulse Transit Time Estimation. Sensors.

[15] Holt C.C. (2004). Forecasting seasonals and trends by exponentially weighted moving averages. Int. J. Forecast..

[16] Yu J., Kim S.B., Bai J., Han S.W. (2020). Comparative Study on Exponentially Weighted Moving Average Approaches for the Self-Starting Forecasting. Appl. Sci..

[17] Lyons R.G. (2010). Understanding Digital Signal Processing.

[18] Makhoul J. (1975). Linear prediction: A tutorial review. Proc. IEEE.

[19] Brennan D.G. (1959). Linear diversity combining techniques. Proc. IRE.

[20] Inan O.T., Kovacs G.T., Giovangrandi L. (2010). Evaluating the Lower-Body Electromyogram Signal Acquired from the Feet as a Noise Reference for Standing Ballistocardiogram Measurements. IEEE Trans. Inf. Technol. Biomed..

[21] Zhang Z., Pi Z., Liu B. (2015). TROIKA: A general framework for heart rate monitoring using wrist-type PPG signals during intensive physical exercise. IEEE Trans. Biomed. Eng..

[22] Koseeyaporn J., Sinchai S., Tuwanut P., Wardkein P. Influence of Light Intensity on a Motion Artifact Signal in a Photoplethysmographic Signal. Proceedings of the 2018 International Conference on Intelligent Informatics and Biomedical Sciences (ICIIBMS 2018).

[23] Bishop C.M. (1995). Neural Networks for Pattern Recognition.

[24] Scardulla F., Cosoli G., Gnoffo C., Antognoli L., Bongiorno F., Diana G., Scalise L., D’Acquisto L., Arnesano M. (2025). Experimental Analysis on the Effect of Contact Pressure and Activity Level as Influencing Factors in PPG Sensor Performance. Sensors.

